# Interleukin-18 modulation in autism spectrum disorders

**DOI:** 10.1186/s12974-015-0466-6

**Published:** 2016-01-05

**Authors:** Rita Businaro, Mariangela Corsi, Gabriella Azzara, Tania Di Raimo, Giovanni Laviola, Emilia Romano, Lidia Ricci, Mauro Maccarrone, Eleonora Aronica, Andrea Fuso, Serafino Ricci

**Affiliations:** Department of Medico-Surgical Sciences and Biotechnologies, Sapienza University of Rome, Corso della Repubblica 79, 04100 Latina, Italy; Section of Department of Cell Biology & Neuroscience, Section Behavioural Neuroscience, Istituto Superiore di Sanità, Roma, Italy; Department of Anatomical, Histological, Legal Medicine and Orthopedics Sciences, Sapienza University of Rome, Rome, Italy; European Center for Brain Research (CERC)/IRCCS Santa Lucia Foundation, Via del Fosso di Fiorano 64-65, 00143 Rome, Italy; School of Medicine and Center of Integrated Research, Campus Bio-Medico University of Rome, via Alvaro del Portillo 21, 00128 Rome, Italy; Department of (Neuro)Pathology, Academic Medical Center and Swammerdam Institute for Life Sciences, Center for Neuroscience, University of Amsterdam, Amsterdam, The Netherlands; Department of Psychology, Sapienza University of Rome, Rome, Italy

**Keywords:** Autism, Cytokines, IL-18, Immunohistochemistry, ELISA, Reeler mice

## Abstract

**Background:**

Autism spectrum disorder (ASD) is a neurodevelopmental disease which affects 1 in 88 children. Its etiology remains basically unknown, but it is apparent that neuroinflammation is involved in disease development. Great attention has been focused on pro-inflammatory cytokines, and several studies have reported their dysfunction unbalance in serum as well as in the brain. The present work aimed at evaluating putative dysregulation of interleukin-18 (IL-18), a pro-inflammatory cytokine of the IL-1 family in the sera of patients with ASD of different grades, compared to healthy controls, as well as in postmortem brain samples obtained from patients with tuberous sclerosis as well as acute inflammatory diseases. Moreover, quantitative analysis of IL-18 was performed in the sera and brain obtained from Reeler mice, an experimental model of autism.

**Methods:**

Serum IL-18 levels were measured by ELISA. IL-18 was localized by immunohistochemical analysis in brain sections obtained from tuberous sclerosis and encephalitis patients, as well as from gender- and age-matched controls, and in the brain sections of both Reeler and wild-type mice. IL-18 was also quantified by Western blots in homogenates of Reeler and wild-type mice brains. IL-18 binding protein (IL-18BP) was evaluated in Reeler and wild-type mice plasma as well as in their brains (sections and homogenates).

**Results:**

IL-18 content decreased in the sera of patients with autism compared to healthy subjects and in Reeler sera compared to wild-type controls. IL-18 was detected within glial cells and neurons in the brain of subjects affected by tuberous sclerosis and encephalitis whereas in healthy subjects, only a weak IL-18 positivity was detected at the level of glial cells. Western blot identified higher amounts of IL-18 in Reeler brain homogenates compared to wild-type littermates. IL-18BP was expressed in higher amounts in Reeler brain compared to the brain of wild-type mice, whereas no significant difference was detected comparing IL-18BP plasma levels.

**Conclusions:**

IL-18 is dysregulated in ASD patients. Further studies seemed necessary to clarify the molecular details behind IL-18 increase in the brain and IL-18 decrease in the sera of patients. An increase in the size of the patient cohort seems necessary to ascertain whether decreased IL-18 content in the sera can become a predictive biomarker of ASD and whether its measure, in combination with other markers (e.g., increased levels of brain-derived neurotrophic factor (BDNF)), may be included in a diagnostic panel.

## Background

Autism spectrum disorder (ASD) is a neurodevelopmental disorder characterized by repetitive and stereotypic behaviors and impairment in social communications. In most cases, ASD is clinically diagnosed during the first 3 years of age and is a lifelong condition for most [1]. The incidence of autism has dramatically increased in recent decades rising from 2–5/10,000 children to 60–100/10,000 [2]. One out of 88 children has been identified with ASD, which marks a 78 % increase since the first report in 2007. A recent systematic review established that evidence for a pro-inflammatory state is stronger for autism spectrum disorders compared to other children and adolescent neuropsychiatric disorders [3]. The etiology of autism is practically still unknown although numerous studies have identified a number of genes and environmental factors related to the development of the disease. Alternatively, the involvement of chronic neuroinflammation has been ascertained [4–7]. Here, dysregulated immune mediators act by altering the normal development of the nervous system leading in particular to the upregulation of inflammatory cytokines in the ASD brain, probably due to altered blood–brain barrier functions [4]. Cytokines have been reported to influence the development of neuronal and glial cells as well as behavioral phenotypes. A bulk of studies showed that multiple members of the large family of cytokines are essential for proper brain development and for synaptic plasticity and responses to injury [5]. Cytokines are produced by neurons, astrocytes as well as microglia, and abnormalities in their levels were reported in association with neurodevelopmental disorders. Interleukin (IL)-6 elevation in the brain, dependent on glia activation, was related to impaired neuroanatomical structures and altered synaptic plasticity. Some cytokines like IL-1*β* and tumor necrosis factor-α (TNF-α) induce neurotoxicity through elevated glutamate production that results in neuronal excitotoxic death [8]. In a previous study, we showed that cytokines IL-1β, IL-6, IL-12, TNF-α, and IL-23 were significantly increased in the blood serum of ASD patients [9]. The chronic alterations in the inflammatory and immunological responses in patients with autism suggest that this can constitute an endophenotype for ASD. Peripheral cytokines are known to affect different behaviors including sickness and depression and are increased in the brain of subjects with Alzheimer’s disease [10, 11]. Since both the neuroinflammatory processes and the increased immune response observed in ASD would comprise high levels of cytokines in the brain, these proteins could affect behavior [12].

Our interest focuses in the present paper on IL-18, a member of the IL-1 family of cytokines, synthesized as an inactive precursor requiring processing by caspase-1 to be activated; IL-18 is associated to several inflammatory disorders influencing both cellular and humoral immunity [13–15]. The activity of IL-18 is balanced by the presence of a high affinity, naturally occurring IL-18 binding protein (IL-18BP). In humans, increased disease severity can be associated with an imbalance of IL-18 to IL-18BP such that the levels of free IL-18 are elevated in the circulation [15]. IL-18 synthesis was demonstrated in different brain regions, mainly at the level of activated microglia; moreover, IL-18 was shown to be increased in the brains of AD patients [16], and in addition, IL-18 was shown to increase amyloid-β production by human neuron-like cells [17] affecting amyloid precursor protein (APP) processing and therefore, Aβ production [18]. Finally, it is know that autism patients exhibit increased amounts of APP in their brain [19].

The aim of the present work is to evaluate IL-18 serum levels in autism patients compared to healthy controls and in the murine experimental model of autism, the Reeler mice, compared to wild-type controls. Furthermore, we investigated the expression of IL-18 in the brain sections obtained from individuals affected by tuberous sclerosis with autistic behavior, mimicking different features of ASD subjects or by inflammatory diseases, compared to normal subjects. IL-18 brain expression was investigated in Reeler and wild-type mice as well.

## Methods

### Autism patients

Twenty-nine patients aged 2–21 including 27 males were recruited for this study at the Center for Autism of Hospital of Chiaromonte/Lagonegro (Potenza) and at the Pediatrics Neuropsychiatry Department of Matera: two Italian regional centers that coordinate services for persons with autism which they were recruited from. Individual clinical characteristics of the 29 patients included in the study are shown in Table 1. All patients present developmental records documenting characteristics and behaviors that met a standardized definition for American Psychiatric Association (ASD; DSM-IV-TR 2000). Nineteen autistic patients were classified as severe, based on a Childhood Autism Rating Scale (CARS) score of 37 or more; three patients were classified with mild-to-moderate disease, as determined by CARS score between 32 and 37; and eight patients were classified as mild, according to CARS score below 32. Twenty-nine healthy age- and gender-matched subjects with no overt neurological or psychiatric abnormalities were selected as the control group.

**Table 1:** Clinical characteristics of the 29 ASD patients

Patient	Gender	Delivery	Babbling (months)	Age first steps (months)	CARS score	Severity	DSM-IV diagnosis	Comorbidities/other relevant clinical data	Family history	Autoimmune diseases allergy
1.	M	Natural	9	16	36.5	Moderate	Autistic disorder		Cardiovascular diseases, diabetes, hypertension, dyslipidemia	Dust (mother)
2.	M	Natural	12	12	56	Severe	Autistic disorder	Impulse dyscontrol disorder	Tumors	Food allergies (mother)
3.	M	Natural	7	20	37	Moderate	Autistic disorder	Inhalant allergens	Diabetes, hypertension, dyslipidemia, neuropsychiatric diseases	Nickel (mother)
4.	M	Caesarean section	6/7	18	32	Mild-moderate	Autistic disorder	Specific phobia	Cardiovascular diseases, diabetes, hypertension, dyslipidemia, tumors, neuropsychiatric diseases	
5.	M	Natural	6	12	45	Severe	Autistic disorder	Allergy to *Dermatophagoides*	Tumors	Psoriasis (mother)
6.	M	Caesarean section	6	12	56.5	Severe	Autistic disorder	Severe language disorder		
7.	M	Caesarean section	6	12	41	Severe	Autistic disorder	Food allergies	Cardiovascular diseases, obesity, diabetes, hypertension, dyslipidemia, tumors, neuropsychiatric diseases	Pollen (mother)
8.	M	Natural	6	24	41.5	Severe	Autistic disorder		Cardiovascular diseases, hypertension, tumors	
9.	M	Natural	6	Severe hypothonia	40	Severe	Autistic disorder		Diabetes, tumors	Psoriasis (mother)
10.	F	Caesarean section	7	24	38	Severe	Autistic disorder		Cardiovascular diseases, obesity, hypertension	Takayasu’s arteritis (mother)
11.	M	Adopted when he was 2 years old			48	Severe	Autistic disorder	Fish and vegetables allergy		Lactose intolerance (mother)
12.	M	Natural childbirth	6 months	18 months	31	Mild	D.P.S. NOS	Intolerant to gluten	Hypertension	Lactose intolerance (mother)
13.	M	Caesarean section	7/8 months	12 months	40.5	Severe	Autistic disorder		Cardiovascular diseases, obesity, Hypertension	Psoriasis (mother)
14.	M	Natural	12 months	12 months	40	Severe	Autistic disorder		Obesity	Celiac disease (mother)
15.	M	Natural	7	12	33	Moderate	Asperger disorder		Cardiovascular diseases, obesity, hypertension, tumors	
16.	M	Caesarean section, Premature, Jaundice	6	14	30.5	Mild	Autistic disorder	Previous hydrocephalus		Rheumatoid arthritis (sister), duster (mother)
17.	M	Natural	30 months	24	48	Severe	Autistic disorder	Discrete obsessive conducted and tendency toward aggression. Cognitive disabilities. Psychopharmacological treatment		Food (mother), drug (father)
18.	M	Natural	30 months	20	38	Severe	Autistic disorder			Food and Drugs (mother and father)
19.	M	Caesarean section	6	17	20	Mild	Pervasive disorder NOS			Atopic dermatitis (brother)
20.	M	Natural	8/9 months	13 months	23.5	Mild	Pervasive disorder NOS		Cardiovascular diseases, obesity, diabetes, hypertension, dyslipidemia	Celiac disease (grand mother), type 1 diabetes (grand mother)
21.	M	Caesarean section	30 months and after regression	13	52	Severe	Autistic disorder		Cardiovascular diseases, tumors	Rheumatoid arthritis (mother), psoriasis (uncle), ragweed hay fever (aunt)
22.	M	Natural	12 followed by regression	19	39.5	Severe	Autistic disorder			Mite allergy (father), type 1 diabetes (grandfather)
23.	M	Natural	6 months	13 months	24	Mild	Pervasive disorder NOS		Cardiovascular diseases, hypertension, tumors	No
24.	M	Caesarean section	6	13	25	Mild	Asperger disorder		Cardiovascular diseases, hypertension, tumors	Oat grass: lactose intolerant (patient), grass allergy (mother, uncle)
25.	M	Caesarean section	12 months	18 months	39	Severe	Pervasive disorder NOS	Dysgenetic pathology ndd; epileptic syndrome pharmacological treatment		No
26.	M	Natural	Not reported	18	46	Severe	Autistic disorder	Epileptic syndrome pharmacological treatment		Psoriasis (father), lactose intolerance (mother)
27.	M	Caesarean section	6	20	51	Severe	Pervasive disorder NOS	Probable dysgenetic pathology ndd; reduced cognitive skills, ongoing antiepileptic treatment	Cardiovascular diseases, hypertension, dyslipidemia	No
28.	F	Natural	6	13	31	Mild	Autistic disorder		Cardiovascular diseases, hypertension, tumors	Grass, mite allergies (father)
29.	M	Natural	15 months	20 months	49	Severe	Autistic disorder	Genetic pathology ndd; moderate-to-severe cognitive disorder	Cardiovascular diseases, hypertension, neuropsychiatric diseases	Nickel allergy (grandmother), psoriasis (mother)

*NOS* not otherwise specified

*ndd* not defined diagnosis

### Anamnestic medical social questionnaire

The questionnaire, conceived and developed by the Operative and Research Unit of Social Medicine (University “Sapienza” of Rome) identifying any phenotypic diversity in autism spectrum, was administrated to the families of patients by the medical staff of the hospital of Chiaromonte/Lagonegro (Potenza) and of the Pediatrics Neuropsychiatry Department of Matera. It was composed of four sections: (i) child’s data including age, weight, height, residence, blood type, age of first diagnosis and the severity of the disease, the type of vaccination, the stages of language development, the sleep patterns and age at weaning, weaning, nutrition, food allergies, and intolerance; (ii) parental data; (iii) the presence of allergies, food allergies, autoimmune diseases and other diseases, type of diet, working site and exposure to possible harmful factors, personal lifestyles, pregnancy and childbirth, previous surgery and family history for major diseases (especially for autoimmune diseases like rheumatoid arthritis, psoriasis, and Crohn’s disease); (iv) socioeconomic aspects, such as the use of computers and mobile phones, services and support provided by local health authorities, town councils, and social and family health associations. The investigation conforms to the principles outlined in the Declaration of Helsinki. Informed consent was obtained before enrollment.

### Blood sample assays

Venous blood from each individual was collected and stored at 4 °C overnight. Serum was obtained after centrifuging at 1500 rpm for 10 min at RT, aliquoted, and stored at −80 °C until use. All the children were medication free and in good physical health at time of blood sample assays.

### Human samples for immunohistochemistry

The cases included in this study were obtained from the archives of the departments of neuropathology of the Academic Medical Center (AMC, University of Amsterdam) and the University Medical Center in Utrecht (UMCU). A total of five brain tissue specimens (cortical tubers) removed from tuberous sclerosis (TSC) patients (age at surgery 2, 3, 3, 7, 47 years; F/M 3/2) undergoing surgery for intractable epilepsy were examined. Tissue was obtained and used in accordance with the Declaration of Helsinki and the AMC Research Code provided by the Medical Ethics Committee and approved by the science committee of the UMC Utrecht biobank subjects. In three of the five cases, information concerning the presence of mild-to-severe autistic behavior was available. Control cortex/white matter was obtained at autopsy from five age- and gender-matched control patients without history of neurological diseases. Furthermore, we also used histologically normal hippocampus (*n* = 3) and specimens from patients with hippocampal sclerosis (*n* = 3) and from two viral encephalitis patients.

Single-label immunohistochemistry with Ab anti-IL-18 (cat no. PAB16177, Abnova; 1:20) was performed, using the Power Vision kit (Immunologic, Duiven, The Netherlands) and 3,3-diaminobenzidine as chromogen [20]; sections were counterstained with hematoxylin.

### Reeler mice

B6C3Fe heterozygous female and wild-type male mice, originally purchased from Jackson Laboratories (USA) were housed in (33 × 13 × 14 cm) Plexiglas boxes with sawdust bedding and a metal top (two animals per cage). Animals were provided with tap water and food pellets (Mucedola, Settimo Milanese, Italy) ad libitum. Mice were kept on a 12-h light/dark schedule (lights off at 06:30 a.m.), and RT was maintained at 21 ± 1 °C with a relative humidity 60 ± 10 %. Mice genotype was determined from tail samples at weaning using the PCR genotyping protocol previously described [21].

A total of 11 WT and 11 heterozygous Reeler mice were used for the present study. All procedures were performed according to European Communities guidelines (EC Council Directives 86/609/EEC and 2010/63/EU) and Italian national legislation on animal experimentation (Decreto L.vo 116/92) and formally approved by the Italian Ministry of Health.

At the age of 1 month, mice were sacrificed under isoflurane anesthesia.

Blood was collected by heart puncture in a test tube containing EDTA 2 g/dL and immediately centrifuged to separate the plasma and then stored at −80 °C. Brains were perfused with PBS, removed, and bisected on the sagittal plane. The left hemisphere was fixed in 4 % formaldehyde for the immunohistochemical assays; the cortex from the right hemisphere was dissected and stored at −80 °C for the bio-molecular assays.

### Reeler brain immunohistochemical analysis

Mice brains were fixed overnight in 10 % neutral-buffered formalin (prepared as follows: formaldehyde 37–40 % 100 ml/L; distilled water 900 ml/L; sodium phosphate, monobasic 4.0 g/L; sodium phosphate, dibasic (anhydrous) 6.5 g/L; Sigma cat. HT50-1-1), dehydrated and embedded in paraffin. Microtome sections (2 μm thick) were stained with hematoxylin-eosin for histological evaluation.

Microtome sections were rehydrated and incubated for 15 min in 0.3 % H_2_O_2_-methanol to block endogenous peroxidases. After extensive washing in PBS, sections were incubated with Ab anti-IL-18 (cat no. PAB16177, Abnova, dilution 1:50), rewashed, and treated with a biotinylated anti mouse antibody and peroxidase-labeled streptavidin (Dako, Glostrup, Denmark). Sections were then incubated with DAB and extensively washed. The specificity of the reaction was assessed by incubating adjacent sections with isotype-matched irrelevant antibodies, instead of the primary antibody. Images were obtained with a Nikon Ni-E microscope (Nikon, Japan). Furthermore, the sections were analyzed by a NIS program for image analysis (Nikon, Japan) to evaluate the intensity of the reaction in different fields. Five different fields were evaluated from each section and an average of 110 cells was counted for each field.

### Western blotting

Brain tissues were isolated and homogenized in lysis buffer (50 mM Tris–HCl, pH 8.0, 150 mM NaCl, 1 % NP-40, 0.1 % SDS) and a mix of phosphatases and proteases inhibitors (Complete Mini, Roche) using an Ultra-Turrax homogenizer at 4 °C. Lysates were sonicated and then clarified by centrifugation for 15 min at 10.000 *g*. Protein concentration was determined using bovine serum albumin (BSA) as a standard in a Bradford reagent assay (Bio-Rad). Total lysates were boiled in SDS sample buffer, separated by SDS-PAGE electrophoresis, and blotted to nitrocellulose, membrane (Bio-Rad). Nitrocellulose membranes were blocked in PBS-Tween-20 (0.1 %) plus 5 % non-fat milk (Fluka) and incubated with anti IL-18 (1:200, cat. PAB16177 Abnova), anti IL-18BP rabbit policlonal (1:250, cat. bs-4040R, Bioss), or mouse monoclonal anti β-actin (1:1000, cod. sc-81178, Santa Cruz Biotech) primary antibodies for 16 h at +4 °C. After washing three times with PBS-Tween, filters were incubated with peroxidase-conjugated secondary antibodies (anti-mouse or rabbit IgG; 1:4000; Amersham) for 1 h at RT. Detection was performed by Enhanced Chemiluminescence kit (EuroClone). For quantitative measurements, Western blot signals were acquired and analyzed by a Fluor-S densitometer and the Quantity One software (Bio-Rad); optical densities (OD) from at least three different experiments were calculated for each sample and normalized with the corresponding β-actin signal OD; the OD ratios were then compared and expressed as the average fold increase, with one (wt control) as the control value. Optical density values of β-actin appeared unaffected by genotype and treatment. Similar results were also obtained with membranes hybridized with pan 14, 3, 3, and β-tubulin antibodies (not-shown; sc-1657 and sc-5274, respectively, both from Santa Cruz Biotech).

### Cytokine production

Levels of serum cytokines were determined by ELISA following the manufacturer’s instructions using the following kits:

- MBL cat 7620 Human IL-18 ELISA kit based on sandwich ELISA sensitivity 120.5 pg/mL;
- Immunological Sciences cat. IK-10144 Human brain-derived neurotrophic factor (BDNF) ELISA kit based on sandwich ELISA sensitivity 15 pg/ml;
- MBL cat 7625 Mouse IL-18 ELISA Kit based on sandwich ELISA sensitivity 25.0 pg/mL;
- MyBioSource cat MBS2507231Mouse IL18BP (interleukin 18 binding protein) ELISA kit based on sandwich ELISA sensitivity 0.094 ng/mL.

### Statistical analysis

Values are expressed as mean ± SD in the text and figures. One-way ANOVA was computed, and Bonferroni’s posttest was used to calculate any significant (*p* < 0.05) difference in this paper.

## Results

Table 1 summarizes clinical characteristics of ASD patients included in the study, describing in detail their intolerances and allergies, their language development, the presence of delay in babbling, CARS score, comorbidities and other relevant clinical data, and family history.

In the present study, focus was placed on the presence of autoimmune disorder, since the other conditions are present in almost comparable levels in the group of healthy patients: maternal autoimmune diseases include psoriasis (four mothers), rheumatoid arthritis, celiac disease, and Takayasu’s arteritis. In one child, we found paternal familiarity for psoriasis. Another child’ sister suffers from rheumatoid arthritis.

**Fig. 1:**
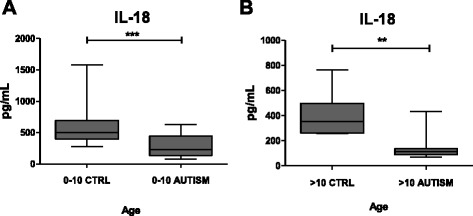
IL-18 serum levels in autism patients and healthy controls. **a** Values observed in autism patients aged below 10 years were significantly lower compared to those of age- and sex-matched healthy controls (*p* < 0.0013); **b** values observed in autism patients above the age of 10 years were significantly lower compared to those of age- and sex-matched healthy controls (*p* < 0.002)

Figure 1 shows the analysis of IL-18 levels in the sera of autism patients and in age- and sex-matched healthy controls. We observed significant differences in the levels of IL-18 present in the sera of autistic patients regardless of age, compared to the values measured in the sera of healthy individuals. Indeed, in patients aged below 10 years, the IL-18 stands at a mean value of 600 pg/mL and goes down to 400 pg /mL in children above the age of 10. The lowest values were observed in patients with severe autism (CARS ≥37).

**Fig. 2:**
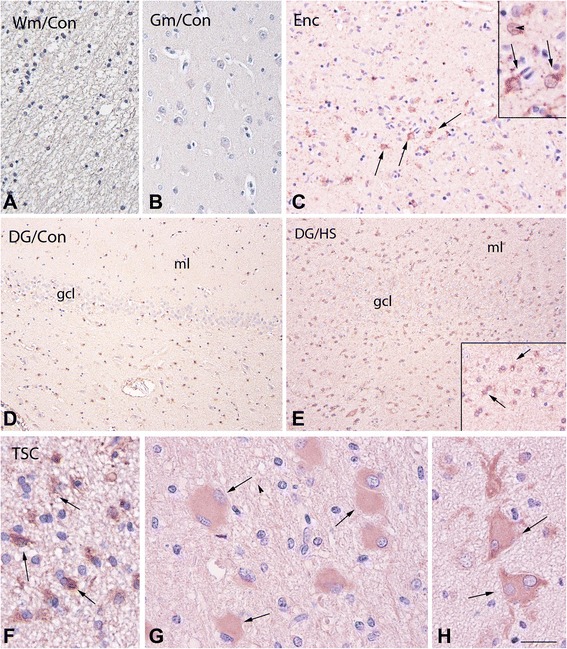
Human brain IL-18 immunoreactivity (IR). **a**–**b** Control white matter (*Wm*; **a**) and gray matter (*Gm*; **b**) showing the absence of detectable labeling. **c** Cortical specimen of a patient with viral encephalitis (herpes simplex encephalitis) with strong IR in astrocytes (*arrows* in **c** and inset). **d**, **e** Dentate gyrus (*DG*) of control (**d**) and hippocampal sclerosis (*HS*, **e**) showing increased expression in HS; inset in **e** shows positive astrocytes; *arrows*); *gcl* granular cell layer, *ml* molecular layer. **f**–**h** TSC specimens (cortical tuber) showing IL-18-positive reactive astrocytes (*arrows* in **f**; *Wm*) and giant cells (*arrows* in **g** and **h**). Microglia in (**e**, **f**). Sections are counterstained with hematoxylin. Scale bar in **h**, **a**–**b**: 80 μm; **d**–**e**: 160 μm; **f**–**h**: 40 μm

In order to check whether a similar difference was also present in the brain, we evaluated the expression of IL-18 by immunohistochemistry in the brain sections (Limbic regions/Frontotemporal cortex + tuber) obtained from patients with encephalitis, tuberous sclerosis, or controls. Tuberous sclerosis is a multi-system genetic disease where 25 to 61 % of affected individuals meet the diagnostic criteria for autism with an even higher proportion showing features of a broader pervasive developmental disorder. As shown in Fig. 2, IL-18 was localized within reactive astrocytes in the hippocampus of patients with medically intractable temporal lobe epilepsy as well as in reactive astrocytes of patients with viral encephalitis and in reactive astrocytes and giant cells in cortical tubers of TSC patients. No reaction (below detection level) or weak positivity at the level of hippocampal astrocytes was present in normal brains.

**Fig. 3:**
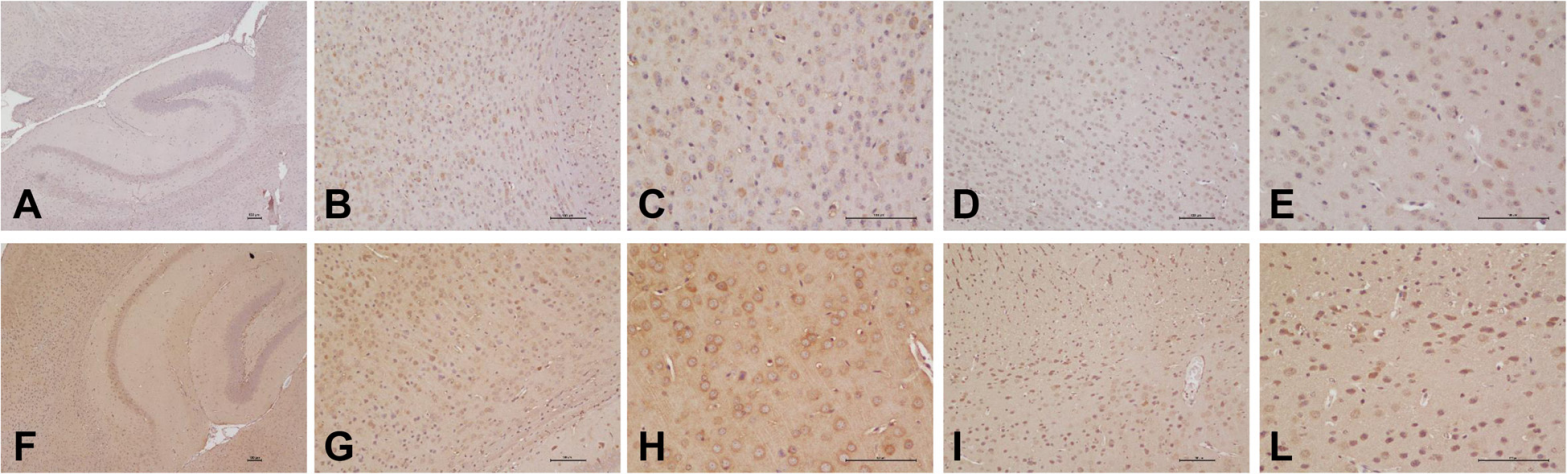
Immunolocalization of IL-18 and IL-18BP in the mice brain. Wild-type mice: IL-18-associated positivity. Neurons as well as astrocytes and microglia are stained. **a** Magnification 4×; **b** magnification 10×; **c** magnification 20×; IL-18BP-associated positivity IL-18. **d** Magnification 10×; **e** magnification 20×. Reeler mice: much neurons and glial cells are stained, and the reaction is stronger. IL-18-associated positivity. **f** Magnification 4×; **g** magnification 10×; **h** magnification 20×; IL-18BP-associated positivity. **i** Magnification 10×; **l** magnification 20×

**Fig. 4:**
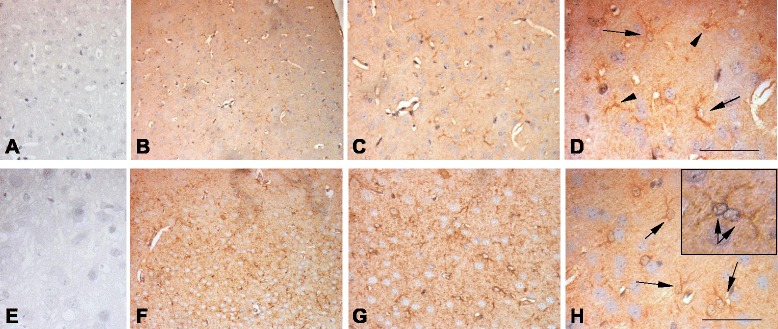
Immunolocalization of IL-18 in the mice brain. Negative control **a** (20×), **e** (40×). The reaction associated to astrocytes and microglia within wild-type brain (**b**, **c**, **d**) and astrocytes and microglia as well as neural cells within Reeler brain (**f**, **g**, **h**). The **h** *inset* depicts positive astrocytes. The positivity was stronger and was detected in higher number of cells within the Reeler brain (**b** 10×, **c** 20×, **d** 40×, **f** 10×, **g** 20×, **h** 40×). Calibration bar 50 μm

**Fig. 5:**
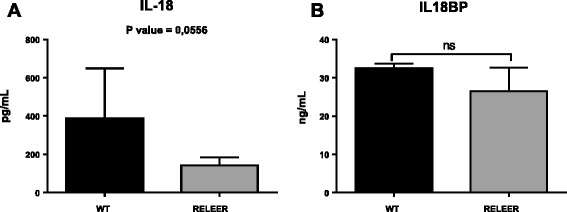
IL-18 and IL-18BP detected by ELISA in the plasma of wild-type and Reeler mice (values expressed as mean ± SD). **a** IL-18 concentrations (pg/mL) and **b** IL-18BP concentrations (ng/mL) in Reeler mice versus wild-type group. Plasma IL-18 was reduced in Reeler mice (*p* = 00.0556). No significant difference was detected in IL-18BP levels in Reeler compared to wild-type mice

**Fig. 6:**
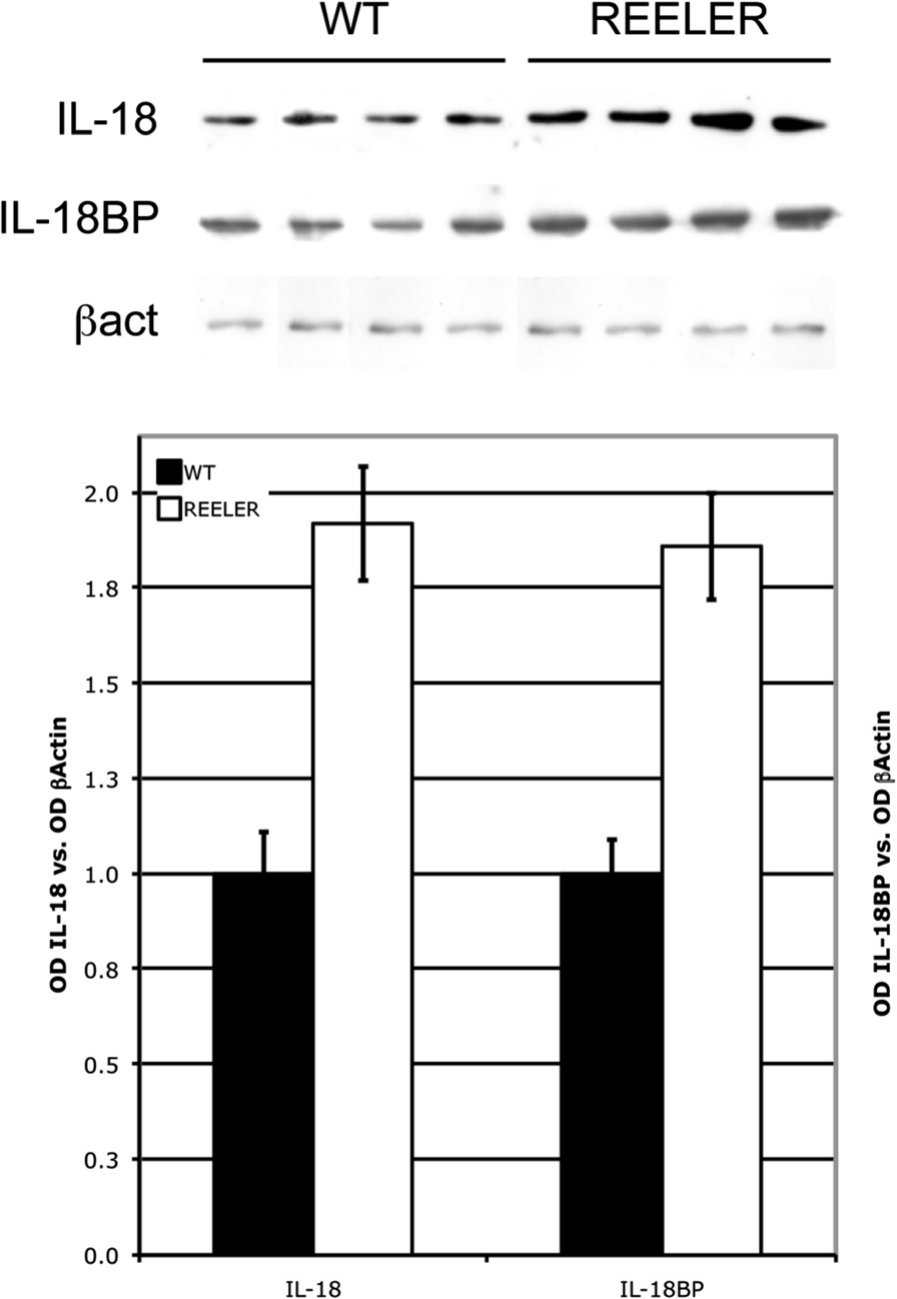
IL-18 and IL-18BP Western blots of mice brain homogenates. Representative Western blot analysis (summarized view corresponding to four animals per group) of IL-18, IL-18BP, and β-actin proteins in cortices of WT and Reeler mice. *Bottom*: semi-quantitative densitometric analysis, obtained by optical density (*OD*) of IL-18 (*left*) and IL-18BP (*right*) normalized with OD of β-actin bands; *N* = 11, *p* < 0.01

We then decided to analyze the expression of IL-18 in the brain of Reeler mice, an experimental model of autism, characterized by a mutation within Reelin, a glycoprotein of the extracellular matrix that plays a key role in migration and positioning of neurons, thus bearing a fundamental neurodevelopmental role in the laminar and columnar organization of the cortex [22, 23]. When levels of Reelin are reduced by 50 %, as in heterozygous mice compared to wild-type ones, several subtle neuroanatomical and behavioral abnormalities are detectable [20]. We studied the presence of IL-18 and IL-18BP in the brain of heterozygous Reeler mice as well as in wild type: results are shown in Figs. 3 and 4 and in Table 2. Several neurons of Reeler heterozygous mice brain were stained, as well as astrocytes, microglia, and giant cells, as shown in Figs. 3 and 4. We quantified the reaction by gray density analysis obtained by NIS-Element AR program and by counting positive cells in five different fields/sections. Reeler brain sections resulted to have higher IL-18 as well as IL-18BP associated positivity (the intensity of the reaction was 10 % bigger; the number of positive cells was tripled for IL-18 and more than doubled for IL-18BP compared to wild-type brain sections). Moreover, we analyzed the presence of IL-18 and IL-18BP in brain homogenates by Western blots. As shown in Fig. 5, immunoblots revealed increased expression of both IL-18 and IL-18BP in the brain of Reeler heterozygous mice compared to wild type. In addition, we measured IL-18 and IL-18 BP levels by ELISA in plasma of both mice strains. The data indicated that IL-18 is reduced in Reeler mice compared to wild type (*p* = 00.0556), whereas there was not a significant difference in the IL-18BP plasma levels from Reeler or wild-type mice (Fig. 6).

**Table 2:** IL-18- and IL-18BP-positive cells in the brain sections

Mice	IL-18-positive cells/total cell number	IL-18-positive cells (%)	IL-18BP-positive cells/total cell number	IL-18BP-positive cells (%)
Wild type	1399/6040	23.16^a^	1477/5905	25.01^a^
Reeler	3417/5655	60.42	4407/6377	69.11

^a^Five different fields were analyzed for each sample (22 samples). In every field, an average of 110 cells was counted *p* < 0.0001

**Fig. 7:**
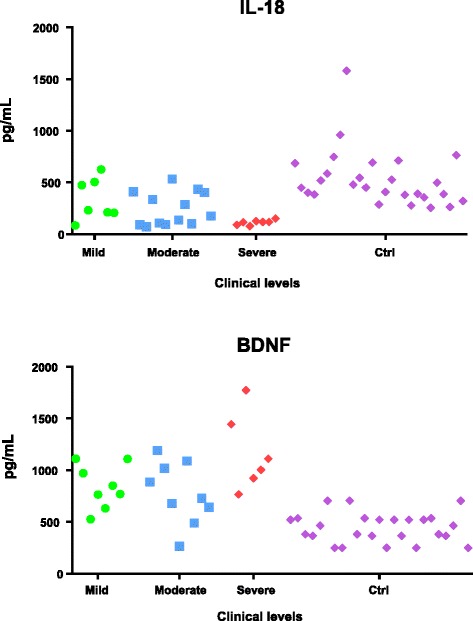
IL-18 and BDNF concentrations (pg/ml) in the sera of autism patients. Autistic patients were classified as severe, based on a Childhood Autism Rating Scale (CARS) score of 37 or more; mild-to-moderate disease as determined by CARS score between 32 and 37; and mild, according to CARS score below 32. An inverse relationship between IL-18 and BDNF was observed in the group of patients with severe autism

Finally, we analyzed BDNF levels in the sera from autism patient cohort. We grouped the patients according to the severity of autism, and as shown in Fig. 7, all three patient groups showed significantly higher levels of BDNF compared to the healthy control group (*t* > 3.34, *p* < 0.01), with higher amounts detected in patients with severe autism (CARS ≥37): severe autism mean = 1170 pg/ml; standard deviation = 372.7; healthy control sample means = 460.4 pg/mL; and standard deviation = 146.78. Patients with severe autism showed IL-18 serum levels lower than the other groups of patients and much lower than the healthy controls: severe autism mean 113.17 pg/mL; standard deviation 23.79; healthy controls mean 529.9 pg/mL; standard deviation = 274.1 (Figs. 1 and 7). Therefore, patients with severe autism (CARS ≥37) show low levels of IL-18 but high levels of BDNF.

## Discussion

Cytokine imbalance was claimed to impact neural activity and mediates behavioral aspects of autism spectrum disorders [24]. ASD is very heterogeneous from a clinical point of view and is equally very heterogeneous on the genetic aspect [25], although a large number of genetic variants associated with ASD converge toward a core set of dysregulated biological processes involving abnormal immune responses. Gene expression studies support the concept that immune and inflammatory genes are upregulated in ASD, a phenomenon observed both in the brain and in peripheral tissues [26]. Furthermore, it is now established that cells belonging to the immune system, such as microglia, are critical to maintaining normal synaptic plasticity and that alterations of these cells may have an impact on neuronal functions [27], and in particular, the cytokines produced by activated microglia have been demonstrated to affect neuronal synaptic function [28]. Previous results showed that neuroinflammation, with increased amounts of IL-1β, IL-6, and TNF-α, takes place in the brains of ASD subjects [29]. We have shown in a previous study that altered levels of IL-1β, IL-6, IL-12, TNF-α, IL-23, and BDNF occur in the sera of autistic patients [9]. The present results indicate that there is also a dysregulation of IL-18 levels, both in the serum obtained from the same group of patients previously under study as well as in brains of TSC affected subjects with an inverse relationship, though, as a matter of fact, we observed IL-18 decrease in the serum and increase in the brain of both ASD patients and mouse model. The largest decrease was detected in the sera of patients with severe autism (CARS ≥37). It is not easy to explain why quantitatively different levels have been found in the brain compared to the sera of individuals with autism. Previous studies reported that plasma levels of IL-18 were significantly elevated in Alzheimer’s disease compared to healthy controls, but that also levels of IL-18 transcript and proteins were increased in frontal lobe of AD patients compared to healthy age-matched controls [16], even if AD-severe patients showed low levels compared to AD-mild patients [30]. El-Ansary and coworkers reported that autistic children exhibited significantly lower serum concentrations of both Aβ (1–40) and Aβ (1–42) compared to control subjects, despite the higher amounts of Aβ found in the brain of the subjects with autism [31]. Moreover, they detected unexpected lower concentrations of caspase-3, TNF-α, and IL-6 in plasma of autistic patients compared to age- and gender-matching controls [32] despite being known from other studies that the same cytokines were increased in the brain of autistic subjects [33]. The gene encoding for IL-18 and its receptor have distinct promoter regions and then alternatively spliced transcripts giving to these molecules the potential to be produced in tissue-/cell-specific way in response to different stimuli [34, 35]. Furthermore, the splice variants of IL-18Rβ chain have been proposed to be the soluble negative regulator of IL-18 action [36, 37].

IL-18 is physiologically produced in different regions of the brain, including the hippocampus, the hypothalamus, and the cerebral cortex, where several isoforms of its receptor have been identified [13]. It has been hypothesized that IL-18 may have a role in central nervous system development and is involved in synaptic plasticity, in glutamate release and enhanced postsynaptic α-amino-3-hydroxy-5-methyl-4-isoxazolepropionic acid (AMPA) receptor responses as well as in fear memory and spatial learning [13, 38]. IL-18 is also induced during physical/emotional stress responses [34, 39] and high levels of IL-18 are detected in several neuropathological conditions such as during microbial infections, following trauma, stroke, or ischemia. Our results, showing higher number of IL-18-positive cells in the brain of viral encephalitis patients confirm these results. In addition, intrathecal injection of IL-18 is able to induce behavioral, morphological, and biochemical changes similar to that detected after nerve injury [40]. Human IL-18 serum levels are elevated in patients with multiple sclerosis [41], Alzheimer’s disease, vascular dementia, and mild cognitive impairment [42]. Previous studies suggested that brain high levels of IL-18 may induce motor and cognitive dysfunctions, impairing learning and memory by acting as an attenuator of long-term potentiation [40, 43]. Our finding is in agreement with previous results detecting increased levels of IL-18 in the brain of an autism experimental mouse model consisting of an inbred strain with behavioral deficits similar to those found in children with autism [44].

In addition, IL-18 can enhance the production of toxic inflammatory molecules such as interferon (IFN)-γ and IL-1β [45, 46], and recent experimental and clinical studies have proven the close connection between the rise of pro-inflammatory cytokines, glucocorticoids, and behavioral changes, such as those associated with anxiety and depression [47, 48]. In this connection, the pro-inflammatory cytokines induce an altered serotonergic function by increasing the conversion of tryptophan to kynurenine. The decrease in the synthesis of serotonin in the brain leads to the formation of neurotoxins such as quinolinic acid and n-methyl-d-aspartate (NMDA) receptor agonist and contributes to increasing apoptotic events in astrocytes, oligodendrocytes, and neurons, exacerbating mood and oxidant status [49]. The diminished serotonin content in the brain of autistic patients was already revealed by positron emission tomography neuroimaging using a serotonin precursor [50] and was related to language and sensory dysfunctions observed in autism [51] as confirmed by the worsening of stereotyped movements observed in autistic children following acute tryptophan depletion and subsequent reduction of serotonin [52]. In fact, it was suggested that autism might be a disorder of serotonin metabolism. Pro-inflammatory cytokines, including IFN-β and IFN-γ, have been shown to reduce the availability of tryptophan, which is required for 5-hydroxytryptamine synthesis through activation of indoleamine-2,3-dioxygenase (IDO), an initiator of kynurenine pathway. IL-18 can enhance production of toxic inflammatory molecules such as IFN-γ [43] and IL-1β [46],which may lead to a vicious cycle where inflammatory processes contribute to different aspects of neurodegeneration. Furthermore, IL-18 belongs to the family of pro-inflammatory cytokines IL-1 and determines an activation signal on neurons and glia increasing both the synaptic release of glutamate and the expression of its postsynaptic AMPA receptor. IL-1β inhibits the removal of glutamate by astrocytes thus causing an excess of this excitatory neurotransmitter that causes neurotoxicity [53].

Further studies are needed to clarify the cause that led to the increase of IL-18 in the brain of patients with autism and its downregulation in sera. We are aware that the number of subjects analyzed is quite small due to the difficulties of collecting samples from autistic children, especially for the postmortem tissues from autistic patients; however, our data represent the first attempt to investigate the role of IL-18 in ASD, and the small sample size seem appropriate for the exploratory aim of this work. Furthermore, increasing the number of cases examined will clarify whether the decrease of IL-18 in sera can be considered a biomarker of the disease and if this measure in combination with other markers, for example, increased levels of BDNF may be included in a diagnostic panel. In addition, the evaluation of SNPs at the level of IL-18 gene or the existence of splice variants for the beta chain of IL-18 receptor proposed to be the soluble negative regulator of IL-18 action may give important information for the better understanding of the mechanisms underlying IL-18 dysregulation.

## Conclusions

Immune dysfunction is present in autism patients. IL-18 is reduced in sera but increased in the brain of patients with tuberous sclerosis with autism. An IL-18 increase was detected also in Reeler brains, mainly at the level of neurons and glial cells; the higher amount of IL-18 was paralleled by a quite similar increase in the amount of IL-18BP. On the contrary, reduced levels of IL-18 were measured in plasma of Reeler mice compared to wild-type mice, whereas no significant variation of IL-18BP was observed. Our data suggest that a chronic neuroinflammation is present in autism affected subjects, including IL-18 dysregulation. The present study might open new scenarios for the comprehension of molecular pathways of the disease.

## Abbreviations

ASD: autism spectrum disorder
IL-18: interleukin-18
IL-1: interleukin-1
BDNF: brain-derived neurotrophic factor
CARS: Childhood Autism Rating Scale
NMDA receptor: n-methyl-d-aspartate receptor
AMPA receptor: α-amino-3-hydroxy-5-methyl-4-isoxazolepropionic acid receptor
